# Reduced heart rate variability during mania in a repeated naturalistic observational study

**DOI:** 10.3389/fpsyt.2023.1250925

**Published:** 2023-09-07

**Authors:** Andrea Stautland, Petter Jakobsen, Ole Bernt Fasmer, Berge Osnes, Jim Torresen, Tine Nordgreen, Ketil J. Oedegaard

**Affiliations:** ^1^Department of Clinical Medicine, University of Bergen, Bergen, Norway; ^2^NORMENT, Division of Psychiatry, Haukeland University Hospital, Bergen, Norway; ^3^Department of Clinical Psychology, University of Bergen, Bergen, Norway; ^4^Department of Informatics and RITMO, University of Oslo, Oslo, Norway; ^5^Division of Psychiatry, Haukeland University Hospital, Bergen, Norway; ^6^Department of Global Public Health and Primary Care, University of Bergen, Bergen, Norway

**Keywords:** bipolar disorder, mania, manic state, heart rate variability, HRV, PPG (photoplethysmography), autonomous nervous system (ANS), vagal activity

## Abstract

**Background:**

Bipolar disorder (BD) is a chronic recurrent mood disorder associated with autonomic nervous system (ANS) dysfunction, indexed by heart rate variability (HRV). Changes in HRV between mood states are sparsely studied longitudinally. We aimed to compare HRV of hospitalized manic individuals with their own euthymic selves in a naturalistic observational study.

**Methods:**

34 individuals were included, of which 16 were lost to follow-up. Ultimately 15 patients provided reliable heart rate data in both a manic and euthymic state, using photoplethysmography (PPG) sensor wristbands overnight. We calculated HRV measures Root Mean Square of Successive Differences (RMSSD), High-frequency (HF: 0.15–0.40 Hz), Low-frequency (LF: 0.40–0.15 Hz), Very low-frequency (VLF: 0.0033–0.04 Hz), Total power and Sample Entropy in 5-min night-time resting samples. We compared HRV measures by mood state within individuals using paired *t*-tests and linear regression to control for age and sex.

**Results:**

HRV was lower in the manic state when compared to the euthymic state for all HRV metrics (*p* ≤ 0.02), with large to medium effect sizes (*g* = 1.24 to 0.65). HRV changes were not significantly affected by age or sex.

**Conclusion:**

This longitudinal study provides evidence of lower HRV in manic states compared to euthymia, indicating an association between ANS dysregulation and changes in bipolar mood state. This corroborates previous cross-sectional studies, although the association may be less clear or reversed in hypomanic states. Further investigation in larger longitudinal samples is warranted.

## 1. Introduction

Bipolar disorder (BD) is a chronic mood disorder characterized by recurrent fluctuations in mood state and energy levels. Although it is a major cause of disability in young adults globally, the mechanisms behind BD remain unclear, and sustaining long-term mood stability is challenging (1). Mood disorders are associated with dysfunction of the autonomic nervous system (ANS), exhibited through reduced vagally mediated heart rate variability (HRV) (2). Previous reviews have described lower HRV in bipolar disorder (BD), indicating an autonomous dysregulation (2, 3). However, findings are inconsistent across studies. The current mood state at data collection is often poorly described. Furthermore, HRV changes between BD mood states are sparsely studied, especially longitudinally (4).

Studying HRV can help us better understand how the heart and ANS respond to environmental changes and stress (5). As BD is characterized by recurring energy and mood changes, corresponding ANS activity changes are anticipated (1). While there is an indication of general ANS dysregulation in BD, the role of the ANS during high-energy manic states is under-investigated. Investigating heart rate variability (HRV) during a manic episode can provide insight into ANS dysregulation and its relation to manic symptoms. Studying HRV longitudinally may provide insight into BD state-dependent changes, as resting HRV typically in relatively stable in healthy individuals (6).

ANS dysregulation has been reported in both bipolar depressed and manic states (2, 4, 7–11). Moreover, two recent studies report an inverse relationship between the severity of previous BD episodes and HRV during euthymia (11, 12). Such previous studies are largely cross-sectional, comparing BD individuals as a group to healthy controls or other psychiatric illness groups (2, 4). Individuals with BD in an unstable phase can be challenging to follow up in longitudinal studies due to the fluctuating nature of their condition and often chaotic life situations. Study participants recruited in one condition, e.g., mania, may lose interest in study participation when transitioned to euthymia or depression, and thereby be lost to follow-up. Nonetheless, studying biological changes between different mood states can provide invaluable information on the biology and etiology of bipolar mood states and is needed (4).

The few available studies that have looked at HRV differences between mood states are small and report conflicting findings. A recent within-individual study of inpatient manic males reported significantly decreased HRV in mania compared to euthymia (13). In contrast, another study reported increased HRV in mild mania compared to euthymia in outpatients (14). These conflicting findings indicate a need for more research on HRV changes between bipolar affective states. In addition to the previously mentioned challenges of longitudinal studies in BD, the lack of HRV studies on multiple affective states might be due to the challenges associated with acquiring electrocardiogram (ECG) data in manic patients. This is particularly due to the elevated psychomotor activity and agitation often associated with moderate to severe mania (15). In this study, we addressed this by utilizing wrist-worn photoplethysmography (PPG) devices to measure heart activity in a closed affective ward. Due to ease of use, this approach can also provide the benefit of a more naturalistic observation compared to conventional ECG recordings in a lab setting. Furthermore, HRV data collected unobtrusively through PPG-wearables is increasingly being proposed as a viable biomarker in BD. Adding a layer of passive objective patient data could greatly aid clinicians in diagnostics monitoring.

### 1.1. Aim

The aim of this study was to compare HRV within hospitalized manic individuals with their euthymic selves in a naturalistic observational study including both sexes. Given the increased energy and agitation associated with mania (15), we hypothesized lower HRV in the manic state compared to the euthymic state.

## 2. Methods

### 2.1. Sample

Eligible probands were individuals with BD with an ongoing manic episode admitted to a closed affective ward at Haukeland University Hospital in Bergen, Norway from November 2017 up to and including May 2020.

Inclusion criteria were Norwegian-speaking individuals 18 to 70 years old diagnosed with BD by the ICD-10 criteria, able to comply with instructions, and cognitive abilities clinically estimated to correspond to an IQ above 70. Participant diagnoses were set or confirmed by resident physicians or senior consultant psychiatrists at Haukeland University Hospital. Exclusion criteria were participation refusal, previous head trauma requiring hospital treatment, an organic brain disorder, pregnancy, ongoing substance dependence (nicotine permitted), and being in a state of withdrawal.

The Norwegian Regional Medical Research Ethics Committee West approved the study (2017/937). All participants gave written consent in accordance with the Helsinki Declaration.

### 2.2. Design and procedure

This study is a within-individual observational case–control study of HRV in bipolar type 1 inpatients. Inpatients were invited to participate upon recommendation from psychiatry residents or senior consultant psychiatrists of closed affective wards at Haukeland University Hospital. No financial compensation or treatment benefits were provided.

**Figure 1:**
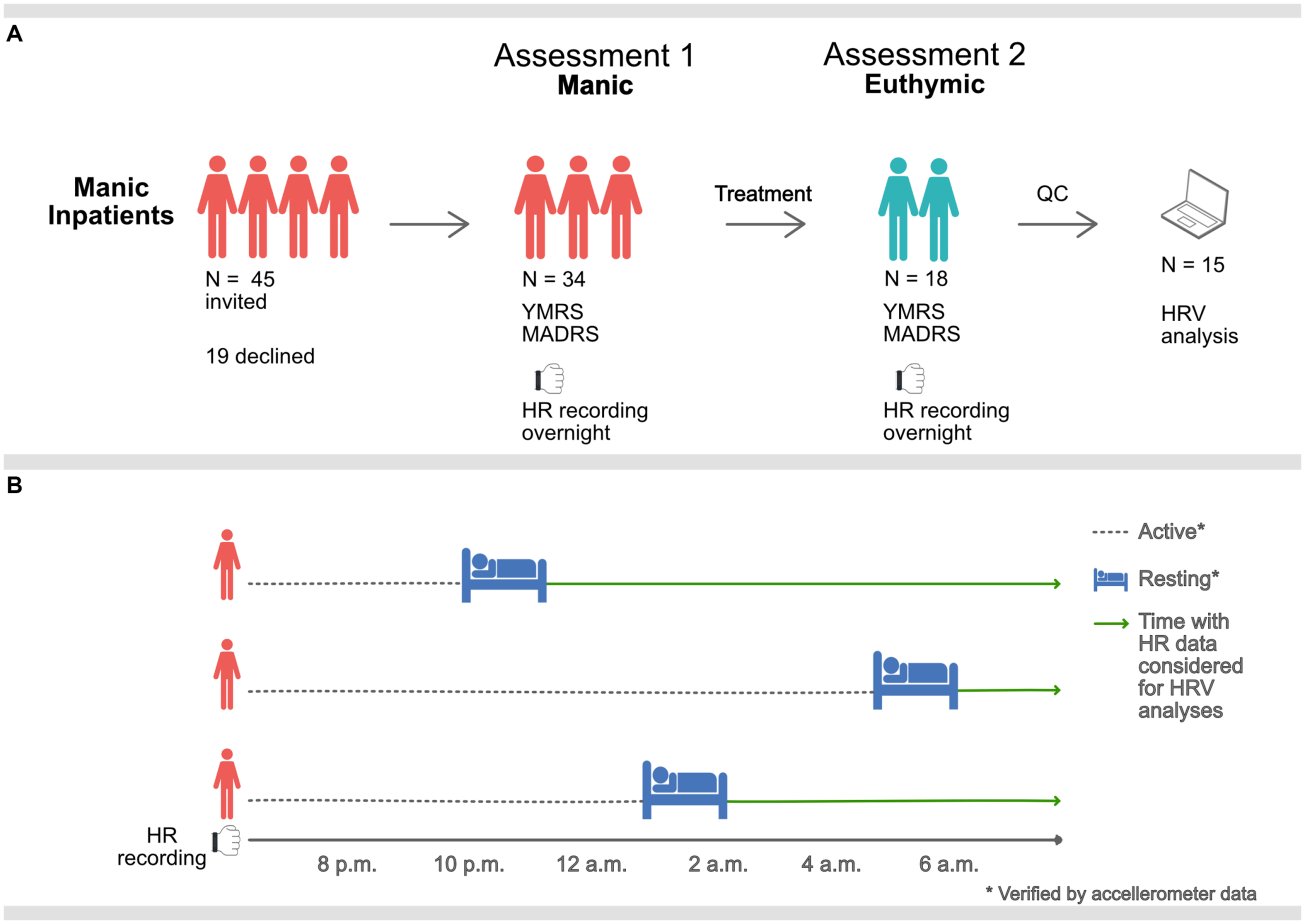
Graphic description of the study procedure. **(A)** Manic inpatients were assessed and equipped with PPG sensors overnight when manic and when euthymic. 15 participants remained after QC of heart rate (HR) data. **(B)** Overnight HR data was only considered for HRV analysis during rest, verified by accelerometer data. Sampling times, therefore, vary (mean 11.34 p.m., standard deviation 112-min).

We assessed the included patients and obtained overnight multi-sensor wristband recordings at the time of inclusion. Participants were reassessed and recorded prior to discharge from the closed ward or at the open ward they were transferred to, when in remission. The study design is presented in Figure 1A.

Behavioral challenges associated with manic states (e.g., motor restlessness, agitation, and psychosis) can make accurate heart rate data collection challenging. The use of an ambulatory device for heart rate sampling was selected to facilitate data collection from a clinically realistic sample in a non-laboratory inpatient setting. Ward routines promote rest and sleep at night, providing a time window for better recording conditions for the recording device (16). Five-minute samples were located at night-time after initiation of long-term rest, confirmed by accelerometer data. Hence, HRV metrics were calculated at unique time points corresponding to the individual’s rest initiation time, see Figure 1B. Due to the differences in motor activity and reduced subjective need for sleep, these resting samples ranged from 8.10 p.m. to 5.10 a.m. (mean 11.34 p.m., standard deviation 112-min). There was no relationship between time of sampling and HRV measures, see Supplementary Table S4.

### 2.3. Measures

#### 2.3.1. Heart rate variability

Wrist-worn Empatica E4 devices with photoplethysmography (PPG) sensors, which have been validated against ECG for HRV purposes, were used for pulse detection (16, 17). Heart rate was monitored overnight in a non-laboratory naturalistic hospital setting at a 64 Hz sampling rate. Research personnel secured the device on the participants’ dominant wrist as tightly as tolerated, ideally to a fit allowing one finger under the band, as recommended by the manufacturer. Participants were instructed not to shower or touch the device during the recording. We used Empatica’s online software, E4 Connect, to visualize the loss of sensor contact, as identified by flattened skin conductance and temperature measurements, and to download the inter-beat-intervals (IBI) data as comma-separated value files for post-processing.

IBI data was analyzed in Kubios HRV Premium software version 3.4.2 (18). Data quality was manually screened, and acceptable data were subjected to an automatic artifact correction threshold and the smoothing priors detrending method (λ = 500), using a five-minute window. We set the artifact correction cut-off to the recommended 5% and considered data with a higher correction rate as poor-quality sections. We excluded recordings from three individuals which did not include any 5-min segments with sufficient data quality throughout the night.

We selected commonly used HRV measures, representing the time, frequency, and non-linear domains. Root mean square of the successive differences (RMSSD), representing the time domain, mirrors the variance in time between heartbeats, is widely used for monitoring vagally mediated (i.e., parasympathetic) HRV changes, and is well-suited for use on our 5-min data segments (19). Total, high frequency (HF), low frequency (LF) and very low frequency (VLF) power represented the frequency domain. High frequency (HF) oscillations (ms^2^/Hz) are often perceived as reflective of predominately parasympathetic influence on the heart and are frequently used in psychology literature (19, 20). This interpretation is controversial, viewed as simplistic, and has received less attention in later years (21). Sample Entropy (SampEn), of the non-linear domain, reflects the complexity and degree of chaos in the heart rate series (19). Finally, high frequency peak values (HF-peak) were used as an indirect measure of respiration frequency (22).

#### 2.3.2. Clinical assessment

The mood state was evaluated using the Young Mania Rating Scale (YMRS) (23, 24) and Montgomery Asberg Depression Rating Scale (MADRS) (25, 26), two commonly used evaluation scales for mania and depression in clinical and research settings. Euthymia was defined as a total YMRS score < 10 (24, 27). BD diagnosis and other psychiatric comorbidities were confirmed by research personnel trained in the use of Mini-International Neuropsychiatric Interview (M.I.N.I.) when the subjects were euthymic (28).

#### 2.3.3. Statistics

We used paired two-tailed *t*-tests to compare HRV between manic and euthymic mood states within the 15 participants. Significance levels were set to *p* = 0.05. The effect size of change by mood state was calculated using Hedges’ g. Sex and age are customary confounders of HRV (19). We ran a separate linear regression model to examine possible confounding effects. The dependent variable of the linear regression models were the manic-euthymic differences of the three HRV metrics and the independent variables were sex and age. Due to the small number of subjects, we did not include mood state as an independent variable in the linear regression models, using the models purely to examine confounding effects. As respiration may confound HRV metrics, we correlated all applied HRV metrics with HF-peak, a proxy measure of respiration (22). Body mass index (BMI) is also known to influence HRV (29). BMI was, however, not available and was not included in the analysis. This is addressed in the discussion. All analyses were performed in the open-source software R using the packages tidyverse, psych, effectsize, and stats base package (30–33).

## 3. Results

### 3.1. Participants

We invited 45 inpatients to participate, of which 34 consented. 18 of the 34 completed both assessment points (manic and euthymic). Ultimately, 15 participants provided recordings that passed quality control and were included in the analysis. See Tables 1, 2 for participant demographics and clinical characteristics.

**Table 1:** Characteristics of *n* = 15 bipolar type 1 study participants at baseline.

*Demographics*	
Mean age (SD)	43 (13)
Range age (minimum – maximum)	21–65
Sex, female, *n* (%)	8 (53)
Marital Status, married/cohabiting, *n* (%)	6 (40)
Employment status, *n* (%)	
Employed/Student	5 (33)
Unemployed	2 (14)
Disability benefits/Retired	8 (53)
Highest level of education, *n* (%)	
Junior high school	5 (33)
High school/Vocational studies	2 (14)
University/Higher education	8 (53)
*Clinical history*	
Depressive episodes, median [IQR]	3 [1–8]
Too many to count/Uncertain, *n*	3
Manic episodes, median [IQR]	4 [3–7]
Too many to count/Uncertain, *n*	3
Age at first manic episode, mean (SD)	30 (10)
Uncertain, *n*	3
Age at first depressive episode, mean (SD)	22 (9)
Uncertain, *n*	6
History of psychosis, *n* (%)	7 (47)

SD, standard deviation; IQR, inter-quartile range.

**Table 2:** Clinical characteristics of *n* = 15 bipolar type 1 study participants during a manic and euthymic state.

*Current episode characteristics*	
Psychopharmacological treatment, (*n*) Manic/Euthymic	
Lithium	4/4
Mood stabilizer	9/9
Antidepressant	2/2
Benzodiazepine	6/3
Sleep aid	4/4
MADRS score, mean (SD)	
Manic state	6 (4)
Euthymic state	5 (4)
YMRS score, mean (SD)	
Manic state	22 (5)
Euthymic state	3 (2)

Antidepressant: vortioxetine. Atypical antipsychotic: aripiprazole, lurasidone, olanzapine, risperidone, quetiapine or zuclopenthixol. Benzodiazepine: oxazepam or diazepam. Non-lithium mood stabilizers: lamotrigine or valproate. Sleep aid: alimemazine or zolpidem. SD, standard deviation; MADRS, Montgomery Aasberg Depression Rating Scale; YMRS, Young Mania Rating Scale.

In accordance with the current recommended treatment of mania requiring hospitalization, all participants used psychotropic medication (1). Medication data were retrieved from patient charts and an overview is presented in Table 2. All in all, medication use was stable between measurement times, although we observed between-state medication changes in three of the 15 participants for both psychotropic and somatic medications. A detailed description of medication use is provided in Supplementary material S2.

### 3.2. Analysis results

**Figure 2:**
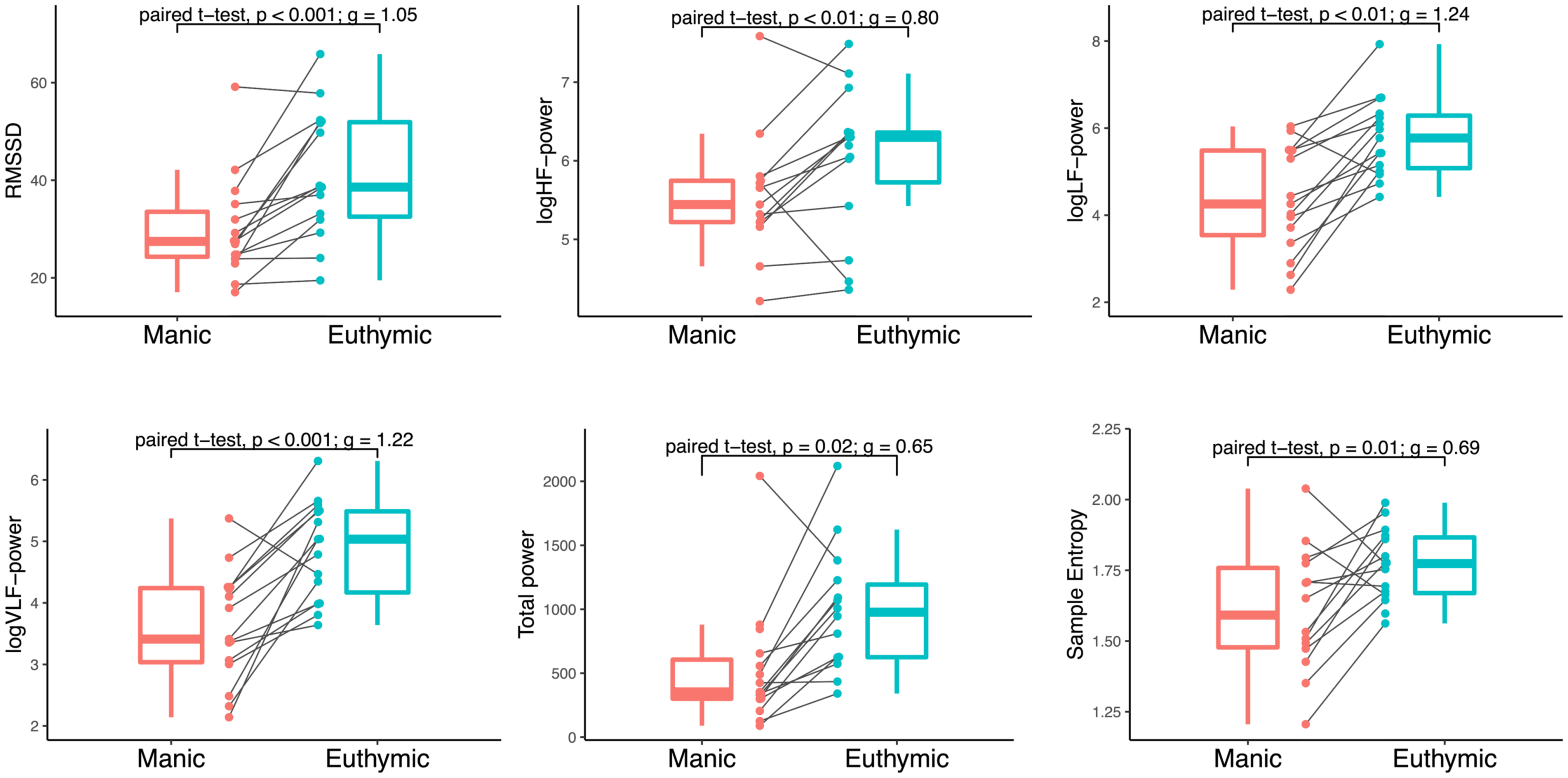
Within-individual comparison of HRV by mood state in 15 bipolar subjects. Paired *t*-tests of RMSSD (ms^2^), logHF-power, logLF-power, logVLF-power [log(ms^2^/Hz)], total power (ms^2^/Hz), and Sample Entropy during mania and euthymia reveal significant differences in HRV between mood states, *p* = 0.05 significance level. Boxplots display median, first, and third quartiles with whiskers extending 1.5 times the interquartile range. The spaghetti plots display HRV metric distribution and development between time points for each participant.

Changes in HRV were observed from mania to euthymia across time-domain, frequency-domain, and non-linear analysis (Figure 2). RMSSD was significantly higher in the euthymic state compared to the manic state (*t* (14)=4.29; *d* = 11.39; 95% CI, 5.70–17.08; *p* < 0.001). The observed effect was large (*g* = 1.05; 95% CI, 0.44–1.71). The same was found for all frequency metrics and SampEn: HF-power (*t* (14)=2.82; *d* = 0.70; 95% CI, 0.17–1.24; *p* = 0.01); LF-power (*t* (14)=5.08; *d* = 1.4; 95% CI, 0.83–2.04; *p* < 0.001); VLF-power (*t* (14)=4.99; *d* = 1.26; 95% CI, 0.72–1.80; *p* < 0.001); total power (*t* (14)=2.65; *d* = 736.08; 95% CI, 140.64–1331.53; *p* = 0.02); SampEn (*t* (14)=3.27; *d* = 0.19; 95% CI, 0.08–0.31; *p* < 0.01), with medium to large effect sizes (HF: *g* = 0.69; 95% CI, 0.14–1.26. LF: *g* = 1.24; 95% CI, 0.59–1.96. VLF: *g* = 1.22; 95% CI, 0.57–1.93. Total power: *g* = 0.65; 95% CI, 0.11–1.21. SampEn: *g* = 0.80; 95% CI, 0.24–1.40). These findings persisted when excluding participants with medication changes (see Supplementary Table S5). The linear regression models revealed no confounding effects of age and sex on any HRV change scores between mood states (see Supplementary Table S3). There were no significant correlations between respiration frequency (HF-peak) and any of the HRV metrics. Of the total sample, one participant showed an opposite pattern in RMSSD, two participants showed an opposite pattern in HF-power, one in LF and VLF-power, and three in SampEn. These participants resembled the overall sample in age, sex, and mean mania baseline and change scores. Two of the three participants with somatic medication changes were among those showing the opposite pattern in LF and VLF and HF and SampEn, respectively.

## 4. Discussion

This study identified a within-individual difference in heart rate variability between the manic and euthymic states in bipolar inpatients. As hypothesized, HRV was significantly lower in the manic state compared to the euthymic state. This is largely in accord with existing cross-sectional studies, which indicate dysregulation of vagally mediated parasympathetic activity during moderate to severe bipolar mania (2, 4).

Our finding of reduced HRV, represented by RMSSD, logHF, and sample entropy, during mania, corroborates previous cross-sectional comparisons of BD mania to healthy controls (2). Robust studies by Henry et al. and Chang et al. have reported reduced HRV variance, HF, and entropy measures in inpatients with moderate bipolar mania when compared to healthy controls (9, 34). Chang et al. also demonstrated an inverse relationship between HRV variance and mania severity, which may indicate a state-dependent relationship between HRV and mood state (9). Overall, our findings on longitudinal HRV changes in BD corroborate previous cross-sectional findings – both in terms of an absolute decrease during mania and the state-dependency of HRV changes. Although HRV changes have been sparsely studied longitudinally, one study used a within-individual design similar to the current study (13). Like us, they found lower HRV in manic BD inpatients compared to their euthymic selves. Unlike our study, which applied ambulatory PPG devices, they used ECG to measure heart rate, included solely males, restricted medication use, and excluded patients with a manic state characterized by high motor activity (13). The current study complements this study, demonstrating that these HRV changes also occur across different anti-manic treatment regimes. Furthermore, while HRV, particularly HF-power, typically differs between sexes, our study found that sex did not influence any of the HRV metrics (see Supplementary Table S3) (35). However, it is not possible to draw conclusions regarding sex differences in HRV within the context of BD due to the study’s limited participant count. Restrictions on sex and motor activity may be related to the use of a chest-strap ECG device, exemplifying the downsides of using the gold standard compared to a wristband PPG.

Our findings are, however, opposed by one longitudinal study of 16 BD outpatients (14). They reported higher HRV in mania compared to euthymia and a positive relationship between HRV and mania scores. The seven participants measured in a manic state were characterized as with mild mania, in contrast to our study sample and those presented above studying moderate to severe mania (9, 13, 14, 34). This could indicate a non-linear progression of HRV changes from euthymia via hypomania to mania, i.e., an initial HRV increase followed by a reduction, and help explain the contrasting findings.

Reduced RMSSD, frequency measures, and SampEn may indicate decreased cardiac vagal influence, suggesting ANS dysregulation (19, 36). The ANS and psychiatric disorders are both complex, and their interaction with brain dysfunction and mania likely involve multiple organ systems and brain regions. The neurovisceral integration model proposes bidirectional links between the heart and prefrontal cortex and subcortical circuitries, relating HRV to adaptability; low HRV is associated with maladaptive self-regulation (20, 37). In line with this model, ANS dysfunction has been proposed as integral to bipolar psychopathology via impaired neural-autonomic coordination in cognitive and emotional processes, making ANS dysfunction a possible target for treatment (38). Furthermore, as ANS disruption occurs not only in mania, but both uni- and bipolar depression, it may reflect a general dysregulation associated with psychopathology (20). Yet, the disruption is greater in bipolar compared to unipolar depression, suggesting a larger role played by ANS dysregulation in bipolar psychopathology (39). In light of these previous findings and theories, the current study reinforces HRV as a potential biomarker for BD disease states. In practice, HRV may facilitate monitoring treatment response and mood stability in BD, given adequate technological advances and further studies.

### 4.1. Limitations

The limitations of sample size, PPG recording challenges, and potential confounders necessitate further investigations to enhance the generalizability of the present findings. The naturalistic observational design provided an ecologically valid sample but posed challenges with compliance, data quality, loss to follow-up, and potentially confounding effects due to medication use. Although the use of PPG sensors allowed for the inclusion of severely affected patients, the recordings were susceptible to movement artifacts and sabotage, resulting in participant exclusion due to poor sensor data quality. Given the circadian abnormalities associated with BD, night-day differences in HRV (diurnal variance) should be explored in future studies, especially as more reliable technological solutions become available. Additionally, these studies should consider examining potential confounding factors such as individual sleep–wake patterns, total motor activity, and nutritional factors. Collecting physiological data from a population in an unstable illness phase is time-consuming and resource-intensive, and previous endeavors have had a similar sample size (13, 14). Hence, BMI, somatic comorbidities, and medications were not controlled for in this study. However, a control analysis excluding participants with medication changes yielded similar results.

The use of a within-subject design, where participants acted as their own controls, increases statistical power and addresses some of the limitations of potential confounders of HRV. Still, moderating effects of variables such as medication use on HRV dynamics within an individual may exist. Nevertheless, the study’s medium to large effect sizes of reduction in HRV within individuals transitioning from a manic to euthymic state indicate interesting results warranting replication in larger studies.

### 4.2. Conclusion

In this repeated naturalistic observational study, we compared HRV in hospitalized individuals with BD in manic and euthymic states. We found lower HRV in the manic state compared to the euthymic state, examining RMSSD, HF power, and Sample Entropy. This decrease indicates dysregulation of the ANS. However, existing literature on the association between HRV and mania is somewhat inconsistent. This study provides evidence that HRV is lower and less complex in manic states, but this association may be less clear or reversed in hypomanic states. Studies in larger samples are needed to examine HRV during the progression to mania via hypomania and investigate moderating effects of medications on HRV changes between mood states.

## Data availability statement

The datasets presented in this article are not readily available because they contain sensitive information on living participants. We have included a minimal dataset of HRV data generated and analyzed in this study, see Supplementary material S1. All data will be made anonymous at the end of the study, November 2025, in accordance with the ethical approval, and be available on reasonable request. Requests to access the datasets should be directed to petter.jakobsen@helse-bergen.no.

## Ethics statement

The studies involving humans were approved by The Norwegian Regional Medical Research Ethics Committee West (2017/937). The studies were conducted in accordance with the local legislation and institutional requirements. The participants provided their written informed consent to participate in this study.

## Author contributions

AS, PJ, OBF, BO, JT, TN, and KJO contributed to this manuscript. PJ, OBF, KJO, TN, and JT contributed to the work’s conception, funding, and design. PJ and AS collected and curated the data. AS and BO analyzed and interpreted the data with KJO. AS wrote the manuscript, which was supplemented and revised by PJ, OBF, BO, JT, TN, and KJO. All authors contributed to the article and approved the submitted version.

## Funding

This work was funded by the Norwegian Research Council (agreement 259293). The funder had no role in study design, data collection, and analysis, decision to publish, or preparation of the manuscript.

## Conflict of interest

The authors declare that the research was conducted in the absence of any commercial or financial relationships that could be construed as a potential conflict of interest.

## Publisher’s note

All claims expressed in this article are solely those of the authors and do not necessarily represent those of their affiliated organizations, or those of the publisher, the editors and the reviewers. Any product that may be evaluated in this article, or claim that may be made by its manufacturer, is not guaranteed or endorsed by the publisher.

## Supplementary material

### SUPPLEMENTARY MATERIAL S1

Minimal data set with output from HRV analyses. De-identified and restricted due to ethical and patient privacy concerns. Data contains analysis results of HRV variables from one manic and one euthymic 5-minute sample from each participant (*N* = 15).

### SUPPLEMENTARY MATERIAL S2

Description of medication use.

### SUPPLEMENTARY TABLE S3

Results from linear regression analyses. Investigating confounding effects of age and sex on heart rate variability (logRMSSD). The dependent variable of the linear regression model was the manic-euthymic differences of the HRV metrics, and the independent variables were sex and age.

### SUPPLEMENTARY TABLE S4

Results from correlation analyses of sampling time and HRV metrics.

### SUPPLEMENTARY TABLE S5

Reults of follow-up paired t-tests of HRV in mania vs euthymia including only participants without medication changes between time points (*N* = 9).

## Abbreviations

ANS: Autonomic Nervous System
BD: Bipolar Disorder
BMI: Body Mass Index
ECG: Electrocardiogram
HF-power: High-Frequency power
HRV: Heart Rate Variability
MADRS: Montgomery Asberg Depression Rating Scale
RMSSD: Root Mean Square of Successive Differences
SampEn: Sample Entropy
PPG: Photoplethysmography
YMRS: Young Mania Rating Scale
